# Supplementary material for: Reference Intervals for Coagulation Parameters in Developmental Hemostasis from Infancy to Adolescence
Source: Diagnostics (Basel). 2022 Oct 20;12(10):2552. doi: 10.3390/diagnostics12102552 (PMC9601585; doi:10.3390/diagnostics12102552)

Supplemental material

Supplemental tables

Table S1. PT: Lower limit (LL, column 2) or upper limit (UL, column 5) of the reference intervals, with 90% confidence interval (columns 3 and 4 for LL and columns 6 and 7 for UL) for each subgroup investigated. d=days, m=months, y=years, F=females, M=males, N=number of subjects in that specific group after outlier exclusion.

group	LL	90% CI		UL	90% CI		N
0-15d	12.6	12.2	12.8	19.7	19.1	20.1	128
F:0-15d	12.6	12.5	12.7	20.4	18.8	21.1	52
M:0-15d	12.6	12.1	13.1	19.5	18.8	19.8	76
15-30d	12.0	11.4	12.3	16.0	15.4	16.4	129
F:15-30d	11.5	11.1	12.3	15.7	15.4	15.8	67
M:15-30d	12.4	12.3	12.5	16.1	15.1	16.4	61
1-6m	11.7	11.5	11.8	15.7	15.3	15.8	255
F:1-6m	11.7	11.4	11.9	15.7	15.2	15.9	112
M:1-6m	11.6	11.4	11.9	15.4	15.0	15.6	142
6-12m	11.8	11.6	12.0	14.9	14.6	15.0	140
F:6-12m	11.8	11.7	12.1	14.8	14.5	15.0	61
M:6-12m	11.5	11.2	11.9	14.9	14.5	15.0	80
1-16y	12.1	11.8	12.3	15.7	15.4	16.1	162
F:1-16y	12.1	11.9	12.5	15.8	15.3	16.1	74
M:1-16y	12.0	11.5	12.4	15.7	15.3	16.1	88
F	11.9	11.7	12.0	16.3	16.1	16.4	363
M	11.8	11.6	12.0	16.4	16.1	16.6	435
all	11.8	11.7	11.9	16.3	16.2	16.5	799

Table S2. PT ratio: Lower limit (LL, column 2) or upper limit (UL, column 5) of the reference intervals, with 90% confidence interval (columns 3 and 4 for LL and columns 6 and 7 for UL) for each subgroup investigated. d=days, m=months, y=years, F=females, M=males, N=number of subjects in that specific group after outlier exclusion.

group	LL	90% CI		UL	90% CI		N
0-15d	0.92	0.90	0.94	1.45	1.40	1.48	128
F:0-15d	0.92	0.92	0.94	1.50	1.38	1.56	52
M:0-15d	0.92	0.89	0.96	1.43	1.38	1.45	76
15-30d	0.88	0.84	0.90	1.18	1.14	1.21	129
F:15-30d	0.85	0.82	0.90	1.15	1.13	1.16	67
M:15-30d	0.91	0.91	0.92	1.18	1.11	1.21	61
1-6m	0.86	0.85	0.87	1.15	1.12	1.16	255
F:1-6m	0.86	0.84	0.87	1.16	1.12	1.17	112
M:1-6m	0.86	0.84	0.87	1.13	1.10	1.15	142
6-12m	0.86	0.86	0.88	1.09	1.07	1.10	140
F:6-12m	0.87	0.86	0.89	1.09	1.07	1.10	61
M:6-12m	0.85	0.82	0.88	1.09	1.07	1.10	80
1-16y	0.89	0.87	0.91	1.16	1.13	1.18	162
F:1-16y	0.89	0.88	0.92	1.16	1.13	1.18	74
M:1-16y	0.88	0.85	0.91	1.16	1.12	1.19	88
F	0.87	0.86	0.88	1.19	1.18	1.20	362
M	0.87	0.86	0.88	1.21	1.18	1.22	435
all	0.87	0.86	0.88	1.20	1.19	1.21	799

Table S3. aPTT: Lower limit (LL, column 2) or upper limit (UL, column 5) of the reference intervals, with 90% confidence interval (columns 3 and 4 for LL and columns 6 and 7 for UL) for each subgroup investigated. d=days, m=months, y=years, F=females, M=males, N=number of subjects in that specific group after outlier exclusion.

group	LL	90% CI		UL	90% CI		N
0-15d	28.8	26.2	30.1	47.8	46.3	48.6	124
F:0-15d	28.5	27.9	30.3	43.5	42.0	43.8	47
M:0-15d	28.2	25.1	30.6	48.1	46.5	49.0	75
15-30d	28.0	27.5	29.1	39.7	38.6	40.7	121
F:15-30d	28.1	27.7	29.4	39.5	38.3	40.1	62
M:15-30d	28.0	27.5	29.8	39.1	37.9	39.8	58
1-6m	26.3	25.6	27.1	39.4	38.5	40.0	240
F:1-6m	25.8	25.1	27.2	38.7	37.3	39.4	102
M:1-6m	26.5	26.0	27.4	38.6	37.5	39.0	134
6-12m	25.1	24.1	25.9	37.1	35.7	37.9	140
F:6-12m	24.9	24.3	26.0	36.2	34.6	37.1	61
M:6-12m	25.8	25.4	26.5	37.6	35.5	38.2	78
1-16y	26.0	25.2	27.3	37.2	36.2	38.1	154
F:1-16y	27.2	26.5	28.4	36.6	35.6	37.4	73
M:1-16y	25.6	24.9	27.2	37.8	36.2	38.5	80
F	25.9	25.2	26.7	39.8	38.9	40.3	347
M	26.1	25.7	26.6	40.5	39.7	40.9	423
all	26.0	25.6	26.5	40.2	39.6	40.6	770

Table S4. aPTT ratio: Lower limit (LL, column 2) or upper limit (UL, column 5) of the reference intervals, with 90% confidence interval (columns 3 and 4 for LL and columns 6 and 7 for UL) for each subgroup investigated. d=days, m=months, y=years, F=females, M=males, N=number of subjects in that specific group after outlier exclusion.

group	LL	90% CI		UL	90% CI		N
0-15d	0.89	0.81	0.93	1.47	1.43	1.50	124
F:0-15d	0.88	0.86	0.93	1.34	1.29	1.35	47
M:0-15d	0.87	0.77	0.94	1.48	1.43	1.51	75
15-30d	0.86	0.85	0.90	1.22	1.19	1.25	121
F:15-30d	0.87	0.85	0.90	1.21	1.17	1.23	62
M:15-30d	0.86	0.84	0.92	1.20	1.17	1.22	58
1-6m	0.81	0.79	0.83	1.21	1.18	1.23	240
F:1-6m	0.79	0.77	0.83	1.19	1.15	1.21	102
M:1-6m	0.81	0.80	0.84	1.19	1.15	1.20	134
6-12m	0.77	0.74	0.80	1.14	1.10	1.17	140
F:6-12m	0.77	0.75	0.80	1.12	1.06	1.14	61
M:6-12m	0.79	0.78	0.81	1.16	1.10	1.17	78
1-16y	0.80	0.77	0.84	1.15	1.11	1.17	154
F:1-16y	0.84	0.82	0.87	1.13	1.10	1.15	73
M:1-16y	0.79	0.77	0.84	1.16	1.12	1.19	80
F	0.80	0.78	0.82	1.23	1.20	1.24	347
M	0.80	0.79	0.82	1.25	1.22	1.26	423
all	0.80	0.79	0.81	1.24	1.22	1.25	770

Table S5. Fibrinogen: Lower limit (LL, column 2) or upper limit (UL, column 5) of the reference intervals, with 90% confidence interval (columns 3 and 4 for LL and columns 6 and 7 for UL) for each subgroup investigated. d=days, m=months, y=years, F=females, M=males, N=number of subjects in that specific group after outlier exclusion.

group	LL	90% CI		UL	90% CI		N
0-15d	161	142	180	481	454	514	122
F:0-15d	159	156	182	492	459	511	48
M:0-15d	167	139	194	440	420	455	73
15-30d	200	173	210	473	438	492	124
F:15-30d	206	203	220	481	435	497	64
M:15-30d	181	154	211	464	423	488	60
1-6m	194	185	200	566	536	584	245
F:1-6m	191	182	200	567	532	585	110
M:1-6m	195	179	208	548	506	576	134
6-12m	175	161	195	463	440	482	136
F:6-12m	181	171	210	464	434	489	60
M:6-12m	169	156	197	484	443	508	77
1-16y	194	184	212	463	443	476	145
F:1-16y	206	189	241	483	444	508	68
M:1-16y	189	179	209	465	439	479	78
F	186	174	197	517	496	531	351
M	186	175	195	485	467	497	419
all	187	178	195	501	486	513	771

Table S6. Thrombin time: Lower limit (LL, column 2) or upper limit (UL, column 5) of the reference intervals, with 90% confidence interval (columns 3 and 4 for LL and columns 6 and 7 for UL) for each subgroup investigated. d=days, m=months, y=years, F=females, M=males, N=number of subjects in that specific group after outlier exclusion.

group	LL	90% CI		UL	90% CI		N
0-15d	14	14	15	21	20	21	117
F:0-15d	15	15	16	20	19	20	42
M:0-15d	14	14	15	21	21	22	73
15-30d	15	15	15	19	18	19	115
F:15-30d	15	15	16	18	18	19	57
M:15-30d	16	15	16	19	19	19	56
1-6m	14	14	14	18	18	19	158
F:1-6m	14	14	14	18	18	19	95
M:1-6m	14	14	15	19	18	19	64
6-12m	14	13	14	19	18	19	89
F:6-12m	14	13	14	19	18	19	58
M:6-12m	14	14	15	19	18	19	31
1-16y	15	15	15	17	17	17	97
F:1-16y	15	15	15	17	17	17	48
M:1-16y	15	15	15	17	17	17	47
F	14	14	15	19	18	19	298
M	14	14	15	19	19	19	267
all	14	14	15	19	19	19	564

Table S7. D-Dimer: Lower limit (LL, column 2) or upper limit (UL, column 5) of the reference intervals, with 90% confidence interval (columns 3 and 4 for LL and columns 6 and 7 for UL) for each subgroup investigated. d=days, m=months, y=years, F=females, M=males, N=number of subjects in that specific group after outlier exclusion.

group	LL	90% CI		UL	90% CI		N
0-15d	0.29	0.27	0.41	1.87	1.71	1.93	78
F:0-15d	0.34	0.30	0.50	1.88	1.57	1.91	28
M:0-15d	0.28	0.27	0.38	1.84	1.60	1.93	50
15-30d	0.27	0.27	0.30	1.12	1.00	1.24	114
F:15-30d	0.28	0.27	0.34	1.04	0.94	1.10	59
M:15-30d	0.27	0.27	0.32	1.31	1.14	1.34	57
1-6m	0.27	0.27	0.27	0.85	0.79	0.88	111
F:1-6m	0.27	0.27	0.27	0.83	0.74	0.85	56
M:1-6m	0.27	0.27	0.28	0.93	0.77	1.00	55
6-12m	0.24	0.21	0.27	0.86	0.82	0.88	67
F:6-12m	0.27	0.27	0.27	0.87	0.83	0.89	50
M:6-12m	0.21	0.20	0.28	0.65	0.56	0.66	17
1-16y	0.27	0.27	0.27	0.54	0.52	0.56	115
F:1-16y	0.27	0.27	0.27	0.60	0.53	0.63	55
M:1-16y	0.27	0.27	0.27	0.52	0.48	0.52	61
F	0.27	0.27	0.27	1.09	0.97	1.18	246
M	0.27	0.26	0.27	1.39	1.31	1.43	245
all	0.27	0.27	0.27	1.21	1.12	1.26	483

Table S8. Factor II: Lower limit (LL, column 2) or upper limit (UL, column 5) of the reference intervals, with 90% confidence interval (columns 3 and 4 for LL and columns 6 and 7 for UL) for each subgroup investigated. d=days, m=months, y=years, F=females, M=males, N=number of subjects in that specific group after outlier exclusion.

group	LL	90% CI		UL	90% CI		N
0-15d	21	17	28	88	81	91	88
F:0-15d	17	16	28	88	75	92	35
M:0-15d	26	24	31	70	62	73	47
15-30d	45	44	49	88	82	90	37
F:15-30d	59	59	62	89	79	90	13
M:15-30d	45	44	49	83	79	83	24
1-6m	46	43	50	103	100	107	130
F:1-6m	49	48	54	103	99	105	59
M:1-6m	44	42	49	109	98	114	72
6-12m	62	61	66	123	114	126	68
F:6-12m	62	62	68	113	108	114	25
M:6-12m	63	61	70	118	107	120	41
1-16y	72	69	76	124	120	129	123
F:1-16y	71	67	78	123	117	124	57
M:1-16y	72	70	77	127	119	132	66
F	38	33	44	122	116	129	191
M	32	29	37	123	117	130	261
all	33	29	37	122	117	125	453

Table S9: Factor V: Lower limit (LL, column 2) or upper limit (UL, column 5) of the reference intervals, with 90% confidence interval (columns 3 and 4 for LL and columns 6 and 7 for UL) for each subgroup investigated. d=days, m=months, y=years, F=females, M=males, N=number of subjects in that specific group after outlier exclusion.

group	LL	90% CI		UL	90% CI		N
0-15d	42	38	50	166	156	169	87
F:0-15d	42	40	59	172	150	179	35
M:0-15d	43	38	51	128	117	131	48
15-30d	58	52	83	196	176	201	37
F:15-30d	85	84	99	199	149	202	13
M:15-30d	55	51	83	187	161	189	24
1-6m	67	63	75	183	174	190	132
F:1-6m	73	64	88	189	175	195	60
M:1-6m	65	63	74	179	168	186	72
6-12m	78	71	88	181	169	189	66
F:6-12m	72	69	94	178	168	180	24
M:6-12m	84	82	92	167	146	171	41
1-16y	77	73	81	157	146	165	122
F:1-16y	77	72	87	157	137	165	57
M:1-16y	76	72	81	157	145	164	65
F	60	49	67	176	170	180	187
M	54	51	61	175	170	183	258
all	57	52	62	175	171	180	445

Table S10: Factor VII: Lower limit (LL, column 2) or upper limit (UL, column 5) of the reference intervals, with 90% confidence interval (columns 3 and 4 for LL and columns 6 and 7 for UL) for each subgroup investigated. d=days, m=months, y=years, F=females, M=males, N=number of subjects in that specific group after outlier exclusion.

group	LL	90% CI		UL	90% CI		N
0-15d	24	21	31	100	92	107	85
F:0-15d	23	20	33	114	99	117	35
M:0-15d	26	22	33	84	78	85	51
15-30d	31	27	47	150	125	157	39
F:15-30d	54	53	68	141	118	142	13
M:15-30d	29	26	45	150	116	157	26
1-6m	43	37	49	141	137	150	133
F:1-6m	48	46	55	141	133	143	61
M:1-6m	38	32	48	145	136	156	72
6-12m	50	47	61	140	133	142	70
F:6-12m	36	34	55	136	130	138	28
M:6-12m	68	65	76	141	132	143	42
1-16y	54	50	62	137	129	140	125
F:1-16y	57	51	66	124	117	127	57
M:1-16y	52	50	62	139	133	141	68
F	35	29	42	140	133	147	196
M	34	31	38	141	137	149	265
all	35	31	39	140	136	142	460

Table S11: Factor X: Lower limit (LL, column 2) or upper limit (UL, column 5) of the reference intervals, with 90% confidence interval (columns 3 and 4 for LL and columns 6 and 7 for UL) for each subgroup investigated. d=days, m=months, y=years, F=females, M=males, N=number of subjects in that specific group after outlier exclusion.

group	LL	90% CI		UL	90% CI		N
0-15d	21	15	28	87	78	93	83
F:0-15d	16	13	27	80	70	83	33
M:0-15d	26	23	31	88	75	94	49
15-30d	38	34	50	112	99	114	38
F:15-30d	53	52	69	114	96	114	13
M:15-30d	36	33	50	101	88	104	25
1-6m	51	48	55	115	108	120	124
F:1-6m	58	57	62	123	109	128	58
M:1-6m	49	47	53	105	96	107	65
6-12m	63	60	68	134	127	136	70
F:6-12m	65	64	73	119	109	121	25
M:6-12m	62	59	69	135	126	137	44
1-16y	62	58	68	119	114	123	121
F:1-16y	65	62	71	116	109	117	56
M:1-16y	60	57	69	118	113	120	64
F	37	35	41	124	116	130	186
M	33	31	35	131	125	136	259
all	34	31	36	130	125	134	449

Table S12: Factor VIII: Lower limit (LL, column 2) or upper limit (UL, column 5) of the reference intervals, with 90% confidence interval (columns 3 and 4 for LL and columns 6 and 7 for UL) for each subgroup investigated. d=days, m=months, y=years, F=females, M=males, N=number of subjects in that specific group after outlier exclusion.

group	LL	90% CI		UL	90% CI		N
0-15d	68	66	79	282	244	296	66
F:0-15d	71	67	96	247	221	250	27
M:0-15d	68	66	79	291	240	298	39
15-30d	74	73	86	218	193	220	29
F:15-30d	86	85	103	159	151	159	9
M:15-30d	74	72	88	219	199	221	20
1-6m	64	57	73	235	217	250	123
F:1-6m	57	53	70	218	197	224	56
M:1-6m	74	72	78	246	216	253	67
6-12m	74	72	81	204	180	212	53
F:6-12m	73	72	83	193	155	200	23
M:6-12m	76	75	90	187	168	192	29
1-16y	66	63	72	207	183	224	107
F:1-16y	64	62	74	179	169	181	46
M:1-16y	68	66	74	216	181	228	60
F	64	58	70	223	209	231	163
M	71	68	74	231	218	241	212
all	68	65	72	225	217	232	374

Table S13: Factor IX: Lower limit (LL, column 2) or upper limit (UL, column 5) of the reference intervals, with 90% confidence interval (columns 3 and 4 for LL and columns 6 and 7 for UL) for each subgroup investigated. d=days, m=months, y=years, F=females, M=males, N=number of subjects in that specific group after outlier exclusion.

group	LL	90% CI		UL	90% CI		N
0-15d	15	13	20	87	76	94	85
F:0-15d	16	15	23	78	69	80	34
M:0-15d	15	13	23	96	80	100	52
15-30d	32	31	40	95	85	97	37
F:15-30d	45	45	52	110	84	112	12
M:15-30d	32	31	40	82	67	84	24
1-6m	36	32	41	110	105	112	131
F:1-6m	36	33	44	108	98	109	58
M:1-6m	35	30	40	107	99	111	71
6-12m	45	34	58	143	137	145	66
F:6-12m	54	53	62	129	107	134	25
M:6-12m	40	32	59	141	132	143	39
1-16y	60	58	62	123	117	127	124
F:1-16y	60	58	65	120	108	125	56
M:1-16y	59	58	62	125	117	128	68
F	24	19	33	123	114	131	190
M	26	22	30	127	119	134	260
all	25	22	29	127	120	132	451

Table S14: Factor XI: Lower limit (LL, column 2) or upper limit (UL, column 5) of the reference intervals, with 90% confidence interval (columns 3 and 4 for LL and columns 6 and 7 for UL) for each subgroup investigated. d=days, m=months, y=years, F=females, M=males, N=number of subjects in that specific group after outlier exclusion.

group	LL	90% CI		UL	90% CI		N
0-15d	14	11	21	86	76	93	83
F:0-15d	14	10	25	85	77	88	34
M:0-15d	15	13	21	69	65	69	48
15-30d	36	33	46	92	82	95	32
F:15-30d	55	55	61	105	83	107	11
M:15-30d	43	42	47	69	63	69	19
1-6m	41	39	44	111	103	114	127
F:1-6m	49	47	53	110	102	112	54
M:1-6m	40	38	43	107	99	114	69
6-12m	68	64	74	149	136	150	60
F:6-12m	72	72	79	130	116	132	20
M:6-12m	73	72	77	126	116	130	35
1-16y	78	69	82	164	157	167	118
F:1-16y	81	79	87	173	163	177	57
M:1-16y	72	63	82	149	143	152	61
F	31	26	40	155	151	157	183
M	26	20	31	154	143	163	248
all	27	23	31	157	152	163	435

Table S15: Factor XII: Lower limit (LL, column 2) or upper limit (UL, column 5) of the reference intervals, with 90% confidence interval (columns 3 and 4 for LL and columns 6 and 7 for UL) for each subgroup investigated. d=days, m=months, y=years, F=females, M=males, N=number of subjects in that specific group after outlier exclusion.

group	LL	90% CI		UL	90% CI		N
0-15d	21	19	26	89	77	94	83
F:0-15d	20	18	30	85	67	89	32
M:0-15d	24	23	27	83	71	86	50
15-30d	20	15	37	109	84	118	32
F:15-30d	47	46	62	81	78	81	9
M:15-30d	18	14	40	113	84	119	22
1-6m	29	22	41	128	121	138	128
F:1-6m	45	44	52	133	119	144	58
M:1-6m	25	20	40	122	112	124	68
6-12m	52	46	69	143	131	151	60
F:6-12m	46	44	69	130	126	130	19
M:6-12m	65	62	77	147	132	152	41
1-16y	67	62	73	147	139	156	116
F:1-16y	73	71	80	151	137	161	55
M:1-16y	60	57	69	146	135	151	62
F	29	23	35	138	131	146	181
M	26	22	30	139	133	147	248
all	27	24	31	138	133	143	429

Table S16: Factor XIII: Lower limit (LL, column 2) or upper limit (UL, column 5) of the reference intervals, with 90% confidence interval (columns 3 and 4 for LL and columns 6 and 7 for UL) for each subgroup investigated. d=days, m=months, y=years, F=females, M=males, N=number of subjects in that specific group after outlier exclusion.

id	LL	90% CI		UL	90% CI		N
0-15d	ND	ND	ND	ND	ND	ND	ND
F:0-15d	ND	ND	ND	ND	ND	ND	ND
M:0-15d	ND	ND	ND	ND	ND	ND	ND
15-30d	ND	ND	ND	ND	ND	ND	ND
F:15-30d	ND	ND	ND	ND	ND	ND	ND
M:15-30d	ND	ND	ND	ND	ND	ND	ND
1-6m	ND	ND	ND	ND	ND	ND	ND
F:1-6m	ND	ND	ND	ND	ND	ND	ND
M:1-6m	ND	ND	ND	ND	ND	ND	ND
6-12m	ND	ND	ND	ND	ND	ND	ND
F:6-12m	ND	ND	ND	ND	ND	ND	ND
M:6-12m	ND	ND	ND	ND	ND	ND	ND
1-16y	67	62	83	179	169	186	87
F:1-16y	85	80	105	185	174	188	39
M:1-16y	65	62	85	164	155	168	47
F	85	80	105	185	174	188	39
M	65	62	85	164	155	168	47
all	67	62	81	179	170	186	87

Table S17: Antithrombin: Lower limit (LL, column 2) or upper limit (UL, column 5) of the reference intervals, with 90% confidence interval (columns 3 and 4 for LL and columns 6 and 7 for UL) for each subgroup investigated. d=days, m=months, y=years, F=females, M=males, N=number of subjects in that specific group after outlier exclusion.

group	LL	90% CI		UL	90% CI		N
0-15d	34	29	39	87	79	92	138
F:0-15d	33	30	42	83	76	86	54
M:0-15d	38	37	41	89	79	94	83
15-30d	51	49	53	100	92	105	125
F:15-30d	53	53	55	104	95	107	64
M:15-30d	44	38	52	92	89	93	62
1-6m	60	57	64	122	117	127	131
F:1-6m	57	54	62	124	117	127	60
M:1-6m	65	62	71	122	114	128	71
6-12m	76	73	82	133	130	135	108
F:6-12m	74	72	82	135	132	136	64
M:6-12m	82	80	88	124	118	128	44
1-16y	88	87	90	122	119	124	138
F:1-16y	90	88	95	124	121	126	66
M:1-16y	87	86	89	118	114	119	72
F	44	40	49	128	124	132	317
M	41	38	45	118	116	121	336
all	43	40	45	124	120	128	653

Table S18: Protein C: Lower limit (LL, column 2) or upper limit (UL, column 5) of the reference intervals, with 90% confidence interval (columns 3 and 4 for LL and columns 6 and 7 for UL) for each subgroup investigated. d=days, m=months, y=years, F=females, M=males, N=number of subjects in that specific group after outlier exclusion.

group	LL	90% CI		UL	90% CI		N
0-15d	20	20	21	53	49	55	63
F:0-15d	22	22	26	52	45	53	22
M:0-15d	20	20	22	53	48	55	41
15-30d	25	23	27	65	63	67	111
F:15-30d	27	25	30	63	59	63	62
M:15-30d	23	21	26	66	58	68	47
1-6m	22	20	26	74	69	76	112
F:1-6m	20	20	24	70	62	75	56
M:1-6m	26	26	29	75	71	77	56
6-12m	48	42	58	116	108	120	67
F:6-12m	61	60	64	111	103	113	44
M:6-12m	47	45	58	118	101	121	21
1-16y	61	58	65	119	113	121	134
F:1-16y	59	57	65	124	118	127	63
M:1-16y	64	62	68	114	109	119	72
F	24	22	27	116	109	122	257
M	23	21	26	109	105	113	240
all	24	21	26	113	108	118	498

Table S19: Protein S: Lower limit (LL, column 2) or upper limit (UL, column 5) of the reference intervals, with 90% confidence interval (columns 3 and 4 for LL and columns 6 and 7 for UL) for each subgroup investigated. d=days, m=months, y=years, F=females, M=males, N=number of subjects in that specific group after outlier exclusion.

group	LL	90% CI		UL	90% CI		N
0-15d	33	31	36	78	73	80	61
F:0-15d	35	35	41	78	72	79	21
M:0-15d	32	31	36	72	67	73	39
15-30d	40	36	44	104	92	110	112
F:15-30d	38	35	45	100	91	106	66
M:15-30d	44	43	48	100	84	108	45
1-6m	49	46	53	119	113	129	114
F:1-6m	47	44	51	133	116	139	59
M:1-6m	56	52	64	114	107	116	56
6-12m	62	59	67	138	128	143	70
F:6-12m	67	65	73	132	121	135	45
M:6-12m	59	58	65	127	119	128	23
1-16y	60	56	65	119	112	125	136
F:1-16y	59	55	67	122	113	128	64
M:1-16y	61	56	67	112	106	115	71
F	41	37	45	126	117	132	257
M	39	36	42	119	114	125	242
all	40	37	43	121	116	126	498

Table S20: VWF:Ag: Lower limit (LL, column 2) or upper limit (UL, column 5) of the reference intervals, with 90% confidence interval (columns 3 and 4 for LL and columns 6 and 7 for UL) for each subgroup investigated. d=days, m=months, y=years, F=females, M=males, N=number of subjects in that specific group after outlier exclusion.

group	LL	90% CI		UL	90% CI		N
0-15d	86	82	103	219	206	226	51
F:0-15d	95	91	129	209	179	212	20
M:0-15d	68	65	96	266	212	271	34
15-30d	96	93	134	226	215	227	19
F:15-30d	139	139	164	194	160	195	4
M:15-30d	96	93	134	226	216	227	15
1-6m	78	69	86	266	244	274	100
F:1-6m	80	78	89	301	285	303	53
M:1-6m	75	66	94	252	219	259	50
6-12m	58	56	67	168	148	171	44
F:6-12m	66	66	70	171	138	172	14
M:6-12m	58	56	73	152	129	156	30
1-16y	54	51	59	169	152	180	123
F:1-16y	52	49	61	176	154	185	58
M:1-16y	56	55	62	160	144	174	65
F	59	52	65	252	226	267	148
M	59	57	65	220	213	225	191
all	60	57	64	243	225	259	343

Table S21: VWF:RCO: Lower limit (LL, column 2) or upper limit (UL, column 5) of the reference intervals, with 90% confidence interval (columns 3 and 4 for LL and columns 6 and 7 for UL) for each subgroup investigated. d=days, m=months, y=years, F=females, M=males, N=number of subjects in that specific group after outlier exclusion.

id	LL	90% CI		UL	90% CI		l
0-15d	49	48	54	138	117	141	39
F:0-15d	53	52	59	176	136	181	16
M:0-15d	49	48	54	109	95	111	24
15-30d	42	42	49	185	132	187	12
F:15-30d	43	43	80	89	80	89	3
M:15-30d	42	42	63	185	134	187	9
1-6m	52	51	55	177	164	180	57
F:1-6m	52	51	57	169	127	175	28
M:1-6m	52	51	57	179	166	181	29
6-12m	43	42	51	123	93	126	22
F:6-12m	42	42	51	124	87	126	8
M:6-12m	52	51	59	88	82	88	13
1-16y	37	37	40	95	91	97	72
F:1-16y	37	37	40	113	89	116	33
M:1-16y	40	37	46	96	91	97	41
F	38	37	42	124	116	126	82
M	43	39	47	112	104	116	107
all	40	38	43	119	112	123	189

Table S22: PTT-LA: Lower limit (LL, column 2) or upper limit (UL, column 5) of the reference intervals, with 90% confidence interval (columns 3 and 4 for LL and columns 6 and 7 for UL) for each subgroup investigated. d=days, m=months, y=years, F=females, M=males, N=number of subjects in that specific group after outlier exclusion.

group	LL	90% CI		UL	90% CI		N
0-15d	35	34	37	54	50	56	53
F:0-15d	31	31	38	50	47	51	18
M:0-15d	35	34	37	55	50	56	36
15-30d	34	33	36	50	48	51	76
F:15-30d	33	33	37	49	48	49	29
M:15-30d	34	33	37	50	48	51	47
1-6m	32	30	33	50	48	51	108
F:1-6m	32	31	34	47	45	48	51
M:1-6m	33	33	35	51	49	51	55
6-12m	32	31	34	47	45	47	78
F:6-12m	32	31	35	46	44	47	42
M:6-12m	32	31	35	46	43	47	35
1-16y	32	31	33	46	45	47	119
F:1-16y	32	31	34	46	44	47	56
M:1-16y	32	31	33	46	45	47	63
F	31	30	33	48	47	49	200
M	32	31	33	50	49	51	237
all	32	31	33	49	48	50	438

Table S23: PTT-LA ratio: Lower limit (LL, column 2) or upper limit (UL, column 5) of the reference intervals, with 90% confidence interval (columns 3 and 4 for LL and columns 6 and 7 for UL) for each subgroup investigated. d=days, m=months, y=years, F=females, M=males, N=number of subjects in that specific group after outlier exclusion.

group	LL	90% CI		UL	90% CI		N
0-15d	0.86	0.85	0.91	1.33	1.24	1.39	53
F:0-15d	0.78	0.76	0.95	1.25	1.18	1.26	18
M:0-15d	0.86	0.85	0.96	1.35	1.24	1.39	36
15-30d	0.83	0.81	0.90	1.23	1.20	1.26	76
F:15-30d	0.82	0.81	0.96	1.21	1.19	1.22	29
M:15-30d	0.84	0.81	0.92	1.24	1.19	1.26	47
1-6m	0.79	0.76	0.82	1.24	1.20	1.26	108
F:1-6m	0.79	0.77	0.84	1.17	1.12	1.19	51
M:1-6m	0.82	0.81	0.86	1.25	1.22	1.27	55
6-12m	0.79	0.77	0.84	1.16	1.11	1.17	78
F:6-12m	0.79	0.77	0.85	1.15	1.09	1.17	42
M:6-12m	0.79	0.77	0.87	1.14	1.05	1.16	35
1-16y	0.79	0.77	0.82	1.14	1.11	1.16	119
F:1-16y	0.79	0.78	0.84	1.14	1.10	1.15	56
M:1-16y	0.79	0.76	0.82	1.15	1.11	1.16	63
F	0.78	0.75	0.81	1.20	1.18	1.22	200
M	0.80	0.78	0.82	1.24	1.21	1.26	237
all	0.79	0.77	0.81	1.22	1.20	1.25	438

Table S24: DRVV Screening: Lower limit (LL, column 2) or upper limit (UL, column 5) of the reference intervals, with 90% confidence interval (columns 3 and 4 for LL and columns 6 and 7 for UL) for each subgroup investigated. d=days, m=months, y=years, F=females, M=males, N=number of subjects in that specific group after outlier exclusion.

group	LL	90% CI		UL	90% CI		N
0-15d	23	23	25	41	39	42	39
F:0-15d	23	23	25	35	33	35	15
M:0-15d	25	25	30	44	40	45	25
15-30d	24	23	26	40	38	40	74
F:15-30d	26	26	28	39	36	39	28
M:15-30d	24	23	26	40	37	41	46
1-6m	24	23	25	37	36	38	119
F:1-6m	24	23	25	37	36	38	58
M:1-6m	24	23	25	37	35	37	60
6-12m	28	27	29	45	43	45	79
F:6-12m	28	27	29	45	40	45	48
M:6-12m	28	28	30	44	42	44	31
1-16y	29	28	30	40	39	41	119
F:1-16y	29	29	30	41	39	41	56
M:1-16y	28	28	30	40	38	40	63
F	25	24	25	40	39	41	205
M	25	24	26	42	41	42	230
all	25	24	25	41	40	42	436

Table S25: DRVV Screening ratio: Lower limit (LL, column 2) or upper limit (UL, column 5) of the reference intervals, with 90% confidence interval (columns 3 and 4 for LL and columns 6 and 7 for UL) for each subgroup investigated. d=days, m=months, y=years, F=females, M=males, N=number of subjects in that specific group after outlier exclusion.

group	LL	90% CI		UL	90% CI		N
0-15d	0.73	0.72	0.78	1.29	1.22	1.31	39
F:0-15d	0.73	0.72	0.79	1.10	1.03	1.10	15
M:0-15d	0.79	0.78	0.86	1.38	1.25	1.40	25
15-30d	0.76	0.73	0.81	1.24	1.19	1.26	74
F:15-30d	0.81	0.81	0.86	1.21	1.14	1.23	28
M:15-30d	0.74	0.72	0.81	1.25	1.18	1.26	46
1-6m	0.75	0.72	0.78	1.16	1.13	1.19	119
F:1-6m	0.74	0.73	0.78	1.16	1.11	1.18	58
M:1-6m	0.75	0.72	0.79	1.14	1.09	1.15	60
6-12m	0.87	0.85	0.90	1.39	1.34	1.42	79
F:6-12m	0.86	0.84	0.91	1.40	1.24	1.42	48
M:6-12m	0.89	0.88	0.92	1.37	1.31	1.38	31
1-16y	0.90	0.89	0.93	1.26	1.22	1.29	119
F:1-16y	0.92	0.91	0.94	1.28	1.22	1.30	56
M:1-16y	0.89	0.88	0.93	1.24	1.20	1.25	63
F	0.77	0.74	0.80	1.25	1.22	1.28	205
M	0.78	0.75	0.81	1.31	1.27	1.33	230
all	0.78	0.76	0.79	1.29	1.26	1.31	436

Table S26: APCR. **Lower limit (LL, column 2) or upper limit (UL, column 5) of the reference intervals, with 90% confidence interval (columns 3 and 4 for LL and columns 6 and 7 for UL) for each subgroup investigated. d=days, m=months, y=years, F=females, M=males, N=number of subjects in that specific group after outlier exclusion.**

group	LL	90% CI		UL	90% CI		N
0-15d	111	98	123	216	207	219	81
F:0-15d	118	116	128	211	191	215	30
M:0-15d	95	88	123	217	206	219	52
15-30d	106	103	113	174	168	176	74
F:15-30d	106	103	123	175	169	176	28
M:15-30d	107	106	114	167	160	169	46
1-6m	95	83	106	182	177	186	119
F:1-6m	106	104	112	182	171	187	57
M:1-6m	74	70	93	182	176	185	64
6-12m	115	113	120	177	170	179	74
F:6-12m	120	119	125	177	166	179	44
M:6-12m	113	112	119	190	171	194	31
1-16y	130	124	137	202	194	205	112
F:1-16y	133	128	146	186	182	187	49
M:1-16y	127	120	137	205	196	209	62
F	112	107	115	191	185	200	211
M	105	96	110	206	199	211	257
all	107	101	111	202	197	207	471

Table S27: APCR ratio: Lower limit (LL, column 2) or upper limit (UL, column 5) of the reference intervals, with 90% confidence interval (columns 3 and 4 for LL and columns 6 and 7 for UL) for each subgroup investigated. d=days, m=months, y=years, F=females, M=males, N=number of subjects in that specific group after outlier exclusion.

group	LL	90% CI		UL	90% CI		N
0-15d	0.76	0.66	0.83	1.47	1.41	1.49	81
F:0-15d	0.80	0.79	0.87	1.44	1.30	1.46	30
M:0-15d	0.65	0.60	0.83	1.47	1.40	1.49	52
15-30d	0.72	0.70	0.77	1.18	1.14	1.19	74
F:15-30d	0.72	0.70	0.84	1.19	1.15	1.20	28
M:15-30d	0.73	0.72	0.78	1.14	1.09	1.15	46
1-6m	0.64	0.56	0.72	1.24	1.20	1.26	119
F:1-6m	0.72	0.70	0.76	1.24	1.17	1.27	57
M:1-6m	0.50	0.48	0.63	1.24	1.19	1.25	64
6-12m	0.78	0.77	0.81	1.20	1.16	1.21	74
F:6-12m	0.82	0.81	0.84	1.20	1.13	1.22	44
M:6-12m	0.77	0.76	0.81	1.29	1.16	1.32	31
1-16y	0.88	0.84	0.93	1.38	1.32	1.39	112
F:1-16y	0.90	0.87	0.99	1.26	1.24	1.27	49
M:1-16y	0.86	0.82	0.93	1.40	1.33	1.42	62
F	0.76	0.73	0.78	1.29	1.25	1.35	211
M	0.71	0.65	0.75	1.40	1.35	1.44	257
all	0.73	0.68	0.75	1.37	1.34	1.41	471

Table S28: the RIs for adults in use in the laboratory

**Factor (unit)**	**Normal range adult**	**Factor (unit)**	**Normal range adult**
PT (s)	11.5-14.5	Factor VIII (%)	52-200
aPTT (s)	26.3-40.3	Factor IX (%)	59-254
Fibrinogen (mg/dL)	200-400	Factor X (%)	53-122
D-Dimer (µg/mL)	<0.50	Factor XI (%)	67-196
Anti-thrombin (%)	80-120	Factor XII (%)	60-150
Thrombin time (s)	<21	Factor XIII (%)	60-150
Protein C (%)	70-130	VWF Ag (%)	55-120
Protein S (%)	74-120	VWF RCO (%)	40-120
Factor II (%)	78-138	APCR (s)	>120
Factor V (%)	78-152	PTT LA (ratio)	0.80-1.20
Factor VII (%)	61-199	DRVV (ratio)	<1.20

Figures S1: Reference intervals for PT in seconds (A), aPTT in seconds (B), PTT-LA in seconds (C), DRVV Screening in seconds (D) and APCR in seconds (E) in the subjects subgrouped by sex and age. The horizontal segments represent the RIs, while the vertical segments indicate the limits of the 90% confidence interval of the RIs. For each class RIs are reported combining males and females (red) or, respectively, only in females (green) or males

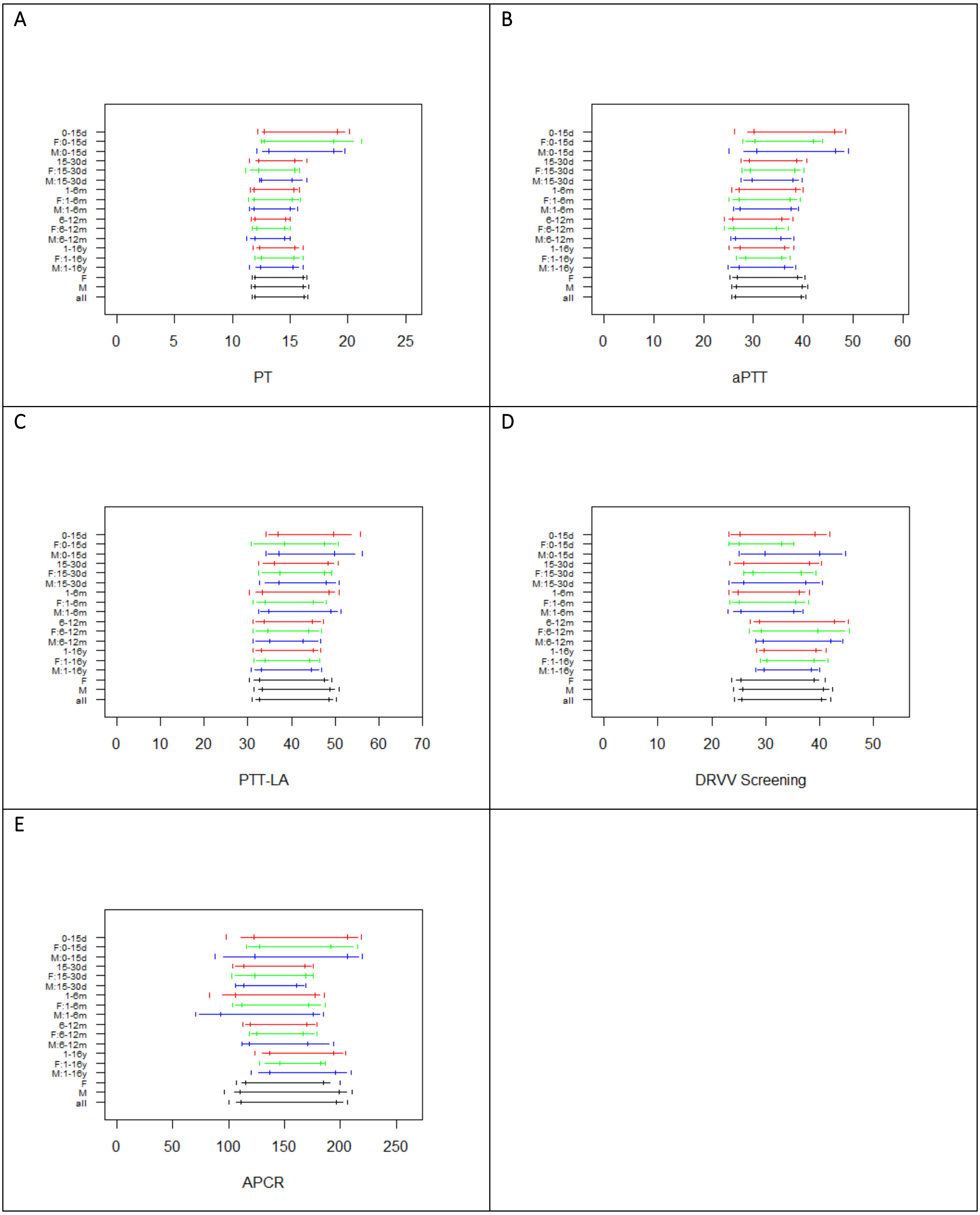

Supplement: Supplementary file 1 [file diagnostics-12-02552-s001.zip › diagnostics-1953215-supplementary.pdf]
